# Fault Recovery Through Online Adaptation of Boolean Network Robots

**DOI:** 10.3390/s25185849

**Published:** 2025-09-19

**Authors:** Paolo Baldini, Michele Braccini, Andrea Roli

**Affiliations:** 1 Department of Computer Science and Engineering (DISI), Alma Mater Studiorum—Università di Bologna, Campus of Cesena, 47521 Cesena, Italy; m.braccini@unibo.it (M.B.);; 2 European Centre for Living Technology, 30123 Venice, Italy

**Keywords:** robotics, fault recovery, fault tolerance, online adaptation, Boolean Networks

## Abstract

Being able to recover from faults is a desired capability in robotics. This requires identifying ineffective behaviors and making some changes so as to display the desired one. In this work, we consider the problem of adjusting the controller of a robot so as to produce the desired behavior. Instead of considering complex and ad-hoc modifications, we leverage the automatic discovery of suitable solutions by means of online adaptation, a mechanism for the modification of the robot control strategy in runtime. Specifically, we use a performance function to identify ineffective behaviors and drive the controller design to an effective one. We also discuss the technical requirements for this procedure to succeed. The results suggest that online adaptation is suitable for the automatic recovery of functions after the occurrence of damages. Additionally, we show that adapting an existing controller to overcome a fault is faster than searching for a new controller from scratch.

## 1. Introduction

The capability to recover and overcome damages is a fundamental ability of living beings [1]. This allows humans, animals, and plants to survive despite damages, recovering or reconfiguring their brain and behaviors so as to overcome them [2]. This greatly increases the chances of individuals’ survival and the continuity of the species. Human-made artifacts have not yet managed to reach a level of autonomy comparable to that of biological systems [3]. Damages are often fatal to human-made products such as robots, requiring manual collection and repair.

Enhancing the autonomy of robots is currently of considerable interest in robotics. Many works tackle the problem of maintaining good behavior in changing contexts [4,5]. Among those stand works on fault tolerance and recovery [6]. We describe fault tolerance as the ability to maintain the desired behavior despite damages, and fault recovery as the modification of some aspects of the robot or controller so as to recover the behavior after the occurrence of damages. For instance, a fault-tolerant robot might rely on redundant sensors so that the occurrence of a single damage would have a reduced impact on the overall control [7]. Differently, a robot displaying fault recovery could overcome issues in movement actuation by decreasing the speed of the movement or by changing its strategy altogether [8,9,10]. The result is that fault tolerance mitigates the impact of the damage but does not necessarily permit to recover the performance. Meanwhile, fault recovery usually displays a more abrupt decrease in effectiveness but, given some time, permits to recover the performance to a level comparable to the original.

In this work, we are more interested in robots displaying fault recovery. In this line of research, previous works have considered the design of an internal state of the robot [9]. When a fault occurs, the system recognizes an unexpected behavior and starts a recovery procedure. This recognizes the new state of the robot and produces a suitable motion plan. Cully et al. [10] created a similar solution, but they stored a library of controllers that the recovery procedure can exploit. More in the line of our work is that of Mahdavi and Bentley [11]. The authors used an evolutionary strategy, which is an optimization algorithm inspired by biological evolution, on a worm-like robot that underwent actuation faults. When damage occurred, the algorithm found new solutions that worked with the current state of the robot. Similarly, Silva et al. [12] modified the controller during operation so as to overcome faults on the actuators, obtaining good results from the point of view of the resilience of the system and its performance. Nevertheless, they did not explore the effect of sensory damages. In Braccini et al. [13], the authors assessed the performance attainable by a robot with different degrees of damages that learned to act online (i.e., in runtime). Nevertheless, their work considered a single task and completely ignored fault identification. Additionally, they did not investigate the advantage of adapting a solution with respect to creating a new one from scratch.

In this work, we consider the use of Online Adaptation (OA), that is, the modification of the control software of a robot in runtime, with the aim of improving a performance measure. We assess whether this induces the recovery of behavioral capabilities affected by the occurrence of different types of faults. Our scenario of investigation is that of minimal robots with constrained computational and power capabilities, with the future goal of developing adaptable miniaturized robots. This limits the possible solutions we can implement, driving the choice to simple computational components with the possibility of realistic hardware implementation. Specifically, we propose to use Boolean Networks (BNs), a model of Gene Regulatory Networks (GRNs). These latter model the interconnection of molecular regulators that governs the expression of genes and have been previously used as controllers for robots [14,15,16]. BNs are indeed dynamical systems with high computational capabilities and possible hardware implementation [17,18], making them suitable for our needs. As a consequence of this research scenario, the adaptive mechanism must also be minimal, implicitly preventing the use of state-of-the-art systems for fault recovery. Herein, we propose a mechanism based on the simple redirection of input signals to different parts of the BN controller, making it easily implementable even in hardware [5]. An embedded evaluation function drives the adaptation, also in this case enabling simple hardware implementation. In this research context, we explore the requirements and limitations for using OA, and we analyze its performance. Additionally, we check the performance drop when the damage occurs and whether the recovery is faster than re-learning a suitable behavior from scratch. The goal is not to produce the best fault-recovery strategy based on OA, but to verify the aforementioned aspects.

This work is organized as follows: Section 2 describes the experiment and the experimental set up. Section 3 presents and explains the produced results. Section 4 discusses the obtained results and draws general considerations on the use of OA for fault recovery. Finally, Section 5 summarizes the work carried out and presents possible future research directions.

## 2. Methods

The goal of this work was to understand if OA can be used for the recovery of robot performance after the occurrence of damages. To this end, we experimented with different types of faults in two tasks and arenas. We employed a two-wheeled robot endowed with proximity and light sensors. The adaptive mechanism used operates on BN-based controllers.

### 2.1. Robot Controller

Generally speaking, in order to perform the desired task, a physical agent needs to act in and react to its environment. The robot perceives the state of the world, and possibly its own state, through the use of sensors. This perception is then processed to produce an action that is expected to help complete the task. Often, this control benefits from the use of internal memory that the robot can exploit.

Among many possible controllers, we proposed one using a BN for its core computation. A BN can be described as a set of interconnected nodes that assume a value in {0, 1} [19,20]. A node value $\nu_{\alpha }$ depends on the value $\nu_{1},\ldots ,\nu_{k}$ of other *k* “input” nodes of the network: $\nu_{\alpha }=f_{\alpha }\left(\nu_{1},\ldots ,\nu_{k}\right)$. The inputs of each node are selected at random among all the nodes of the BN. This creates internal loops that enable storing information like a memory. Indeed, the system evolves in time according to its own past state, implicitly storing information and displaying some sort of fading memory [21,22]. Potentially, a node value could depend even on its own state at previous iterations. Nevertheless, in this work, we prevented this situation due to the peculiar effects it induces in the computation [23].

After deciding the topology of the network (i.e., the *k* inputs for each node), it is important to decide the update function. This maps each possible state of the inputs to the new state of the node, and it is usually represented as a transition table. The update function can be the same for every node, or specific to each one. Herein, we used the latter approach, as for every node, the outputs of the transition table were generated at random according to a bias *p*. Specifically, for a given input state, we selected the resulting output at random: 1 with probability *p*, and 0 with probability 1−p. The update of the state of the BN can be synchronous or asynchronous. In the first case, all the nodes update according to the state of the network at a previous step. In the second case, the nodes update asynchronously according to the current state of the network. In this work, we used synchronous BNs. Additionally, we selected *k* and *p* so as to maximize the computational capabilities of the network—3 and 0.79, respectively [24]. Indeed, *k* and *p* determine the dynamic behavior of the BN and its information processing and storing [21,25,26,27,28]. Finally, we used a BN with 500 nodes.

One obvious limitation of BNs is that they are not designed to work with non-Boolean values. This means that in order to work with analog values such as those produced by robot sensors, they first need to be converted. Many approaches exist to feed non-Boolean values into a BN. For instance, one could feed the digital representation of a number to different nodes of the network. Alternatively, this digital representation could be fed into subsequent time steps. In this work, we used a simple approach that consisted in binarizing values according to a threshold. Specifically, if the perceived value was greater than 0.2, it was converted to 1; otherwise, it was converted to 0. Similarly, the state of a BN can be re-converted to non-Boolean values using strategies opposite to those presented earlier. For instance, the state could be interpreted as a digital representation of a number. Alternatively, the succession of values of a node in time could be averaged or, again, considered as the digital representation of a number. In this work, we selected the values of two BN nodes and multiplied them by 2 so as to control the actuators [13]. Therefore, the control value for the motors of our robot was in {0, 2}.

### 2.2. Adaptive Mechanism

The binarized sensory inputs and the control values for the actuators were, respectively, the inputs and outputs of the BN controller. Specifically, the former overwrote the values of some selected nodes, while the values of other nodes controlled the latter. We call each of these input-to-node and node-to-output mappings a “coupling” [29].

At the start of the experiment, the robot selected a random set of couplings, with the constraint that no coupling insists on the same BN node. This guaranteed that the sensors did not overwrite each other value, and that the outputs did not take the value of an input directly. Throughout the experiment, the robot underwent some adaptation phases in which the couplings changed. Specifically, this mechanism re-coupled up to six inputs of the best-known couplings set to different BN nodes. If the new set of couplings attained better performance than their original set, it became the new best set and the starting point for subsequent adaptations. The output couplings remained unmodified throughout the whole experiment.

In an offline setting, the fastest way to select a controller for the robot is to try different solutions and to select the best. This is possible because the testing environment is predefined and does not usually change during the design. This provides knowledge about the global performance of the robot, reducing the influence of situational conditions on the evaluation [30]. However, in an online setting such as the one we considered, it is also important to maintain the performance of the robot in time [5]. This means somehow balancing exploration and exploitation of the best controller found. In this work, we employed the simple strategy of alternating exploration and exploitation. We did not aim to devise the most effective or balanced strategy for orchestrating these two phases, but rather to present a clear approach to proceed with our investigations. The robot performed an explorative step immediately followed by an exploitative one, thus avoiding extended periods of underperformance while still allowing it to explore. During exploitation, the robot used the best controllers it knew. Nevertheless, over time, these could have stopped being effective due to changes in operational conditions. For instance, the external environment or the robot itself could have changed, making the controller ineffective. To face this kind of situation, the robot continuously updated the performance of the best known controller. Specifically, it averaged the previous performance with the new one. Over time, this reduced the performance of the controller previously considered the best (but that was no longer effective), enabling a better one to replace it.

### 2.3. Tasks

In this work, we considered two frequently used tasks [31]: (i) collision avoidance and (ii) phototaxis. While performing collision avoidance, the robot had to move as fast and straight as possible while avoiding collisions. The evaluation function, therefore, had to consider the maximum proximity to obstacles perceived, as well as the speed and direction of the movement. To compute the performance of the robot in an epoch, we employed the function proposed by Floreano and Mondada [32] and used in many other subsequent works [33,34]:

(1) $$\mathrm{perf}\left(e\right)=\sum_{\;i}^{\;N}\;\left(1-prox_{max,\;i}\right)\times \left(\frac{a_{l,\;i}+a_{r,\;i}}{2}\right)\times \left(1-\sqrt{|a_{l,\;i}-a_{r,\;i}|}\right)$$

where:

- *e* is the epoch under evaluation;
- *i* is the index of a step in the epoch;
- *N* is the number of steps in the epoch, equal to 1500 (i.e., 150 s);
- $prox_{max,\;i}$ is the maximum proximity perceived by the robot, in [0,1], in step *i*;
- $a_{l,\;i}$ and $a_{r,\;i}$ indicate whether the left and right motors of the robot are active in step *i*, with 0 indicating a still motor and 1 indicating a turning one.

**Figure 1:**
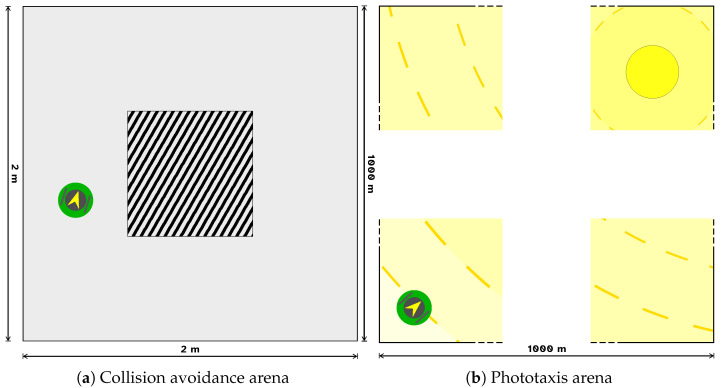
Representation of the arenas used in the experiment. (**a**) The arena for the collision avoidance task. The block in the center represents an obstacle to be avoided. (**b**) The arena for the phototaxis task. The yellow circle represents the light source, and the concentric lines starting from it represent the gradient of luminous radiation. As the arena is too big to be fully represented, here we just show its main parts. Both arenas contain a green entity representing the robot.

We experimented with the collision avoidance task in a squared arena with a central block (see Figure 1a). The robot started in a random position of the arena and had to travel the circuit as fast as possible in a straight line. The best strategy consisted of going straight in the side corridors and turning as fast as possible at the corners.

The second task that we considered was phototaxis. The robot had to follow a light gradient to get as near as possible to its source. This required using light sensors to perceive the signal and wheeled motors to move toward it. We calculated the quality of the behavior in function of the distance traveled towards the light. Specifically, we simply evaluated the performance of the robot as the difference between the distance from the light at the beginning and at the end of an evaluation epoch, as described by the following function:

(2) $$\mathrm{perf}\left(e\right)=D_{start,\;e}-D_{end,\;e}$$

where:

- *e* is the epoch under evaluation;
- $D_{start,\;e}$ is the distance of the robot from the light source at the start of the epoch;
- $D_{end,\;e}$ is the distance of the robot from the light source at the end of the epoch.

This is a proxy for the equivalent local evaluation function employing the light sensors to understand the distance. Indeed, the robot can understand if it is moving in the correct direction and the distance from the light thanks to changes in its perception. An increase in the maximum perceived intensity indicates a movement toward the light, while a decrease indicates that it is going away from it. Similarly, the change in the radiation intensity between the start and the end of an epoch indicates the distance traveled toward the light. This evaluation function is conceptually similar to functions used in other works [33,35].

An epoch of the phototaxis task is composed of 450 steps, for a total of 45 s. We experimented with the phototaxis task in a long empty arena with a light source in the top-right corner (see Figure 1b). The robot always started in the bottom-left corner, which was the furthest from the light, and had to get as near as possible to it. We made sure that the robot sensors perceived the luminous radiation in any point of the arena. The size of one side of the arena was 1000 m, permitting the robot to move without ever reaching the luminous source during the duration of the experiment. This avoided a condition in which the robot could not achieve a positive performance due to having already reached the source.

### 2.4. Damage Types

**Figure 2:**
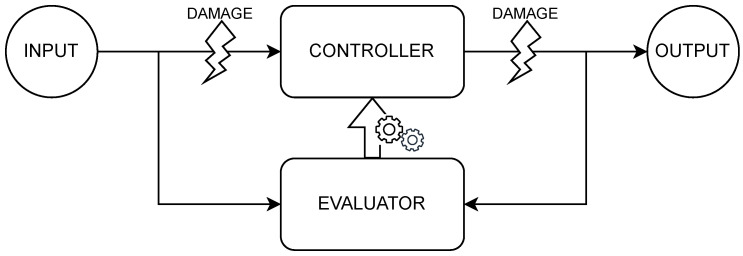
Representation of the points of occurrence of the damages considered in this work.

In this experiment, we explored the capability to recover from different types of faults. Specifically, we imagined issues with both perception and actuation so as to mimic a realistic scenario. Nevertheless, the need to perform self-evaluation to drive the OA required us to focus on internal errors, which are errors occurring on the connection between sensors/actuators and the controller of the robot (see Figure 2). These could undermine the robot behavior but still permit self-evaluation and thus adaptation. The first type of damage we considered was on the wheeled motors. Obviously, zeroing the ability to move would completely impair the robot. Therefore, we imagined a softer damage that just slowed down the motors speed. For instance, imagining a rotation speed of *X* rad/s without damage, its speed while damaged would be 0.5·*X* rad/s. The result is that the robot moves slower on the side presenting the fault, modifying its trajectory and speed. We tested if the robot was able to maintain the correct behavior with zero, one, or both motors functioning at half of their original speed.

The other types of damage that we explored affect the connection of the sensors to the controller. Specifically, we assessed the effect of (i) blind (or missing) perception, (ii) fixed perception, and (iii) random perception. Blind perception occurs when the sensor–controller connections fail, becoming unable to forward the sensory signal. Practically, we implemented this by avoiding overwriting the BN input nodes with the perceived signals. This reduced the information available to the controller, making it partially blind and damaging the feedback loop. Meanwhile, fixed perception occurs when the overwriting process of BN input nodes blocks. We implemented this by selecting a random value and by continuously feeding it to the BN controller. Even in this case, the robot became partially blind to the external world. Finally, we imagined the sensor–controller connection to behave erratically, perturbing the BN controller with random values.

All these types of sensory faults represent possible real-world issues. Whatever the problem, they make the robot at least partially blind to the real world. Nevertheless, there are motivations to assess the effect of each of them separately. Indeed, the management of each type of damage might differ, with some being more complicated to overcome. For instance, blind perception removes information from the computation, but it does not negatively perturb the BN. Fixed perception affects the computation but can be easily nullified by connecting it to a node that ignores its (fixed) value. Again, it can even be used to favorably modify the dynamics of the BN. Finally, random perception might have the biggest impact, requiring one to ignore the signal regardless of its value. Therefore, we assessed the effect of all these types of sensory faults. For each, we tested the effect of a different number of damaged sensors, starting with 0 and increasing the number by 3 up to a total of 24: 0, 3, 6, …, 21, 24. All the faults occurred on contiguous sensors so as to mimic localized faults.

### 2.5. Experimental Setting

All the experiments of this work used a foot-bot [36,37], which is a two-wheeled robot endowed with multiple sensory capabilities. In this work, we used just two of them: proximity sensors for the collision avoidance task and light sensors for the phototaxis task. The robot has 24 sensors for each of these two types, distributed homogeneously around the chassis. The robot acts by means of two differential steering wheels, whose speed we limited to 2 cm/s. All the experiments took place in a simulated environment powered by the ARGoS3 simulator, beta 48 [38].

For each type and intensity of damage, we tested 2500 robot controllers that represented our replicas of the experiment. The controllers were all different inside the same experiment but were the same across different ones. For instance, replica 3 from the experiment with 6 blind sensors had the same starting controller as replica 3 from the experiment with 24 fixed sensors. This guaranteed that the experiments were comparable. After running the experiments, we filtered out the replicas in which the robot did not learn the behavior without damages. Indeed, being able to complete the task is a necessary condition for discussing fault recovery. Additionally, since the controller of the robot used a random BN with fixed output couplings, it is possible that for some replicas, a working controller did not exist at all. This might be due to topological pathologies or to the connection of the motors to fixed output nodes. The result is that for these pathological cases, the adaptive mechanism that we employed was inadequate. In order to avoid considering these situations, we set a threshold for each task discriminating between robots that learned the behavior and robots that did not. We selected the thresholds considering the characteristics of the evaluation functions and empirically observing multiple runs. Specifically, in the collision avoidance task, a performance greater than 500 indicates the ability to avoid obstacles, even though not necessarily efficiently. Just imagine a robot traveling a straight corridor at its maximum speed. This is the only condition in which it could attain the maximum performance: 1500. In this situation, a robot constantly perceiving an obstacle at 1/3 of its perception distance would obtain a performance of 500. Meanwhile, a robot attaining the maximum step performance for 1/3 of the evaluation epoch would also attain a performance of 500. Both situations thus represent a reasonable behavior. Furthermore, both assume that the robot follows a straight trajectory at its maximum speed, which is not a reasonable assumption. This means that a robot attaining a performance of 500 would likely avoid obstacles quite effectively. Therefore, we state that the selected threshold is theoretically sound for the identification of effective behaviors in the collision avoidance task. We confirmed this choice after visual inspection of some runs. In the phototaxis task, we selected 0 as the threshold, identifying robots that averagely move toward the light during the experiment. This is because the evaluation function for the phototaxis task represents movements toward the light as positive values and movements away from it as negative values. Higher performance indicates more effective behaviors. We considered only replicas whose average performance during exploitation epochs (i.e., the ones using the best behavior known) in the first phase of the experiment (i.e., without damages) was higher than the threshold.

In this work, we were also interested in the recovery time of the performance when adapting a previously working controller. To this end, we decided to compare the results to those of a controller designed from scratch for the damaged robot. We refer to the first set of experiments as “informed” and to the second as “clueless” so as to represent the ability of the robot when damage occurs. Clueless robots started in the same position as the informed ones when damage occurred. Additionally, we provided clueless robots the same time to adapt to damages as informed ones. Specifically, informed robots had 480 epochs to learn the behavior and another 480 to adapt it to overcome the faults. Clueless robots had 480 epochs to develop a controller from scratch considering the damages.

All the code used in this work and the produced data are available online [39,40].

## 3. Results

**Figure 3:**
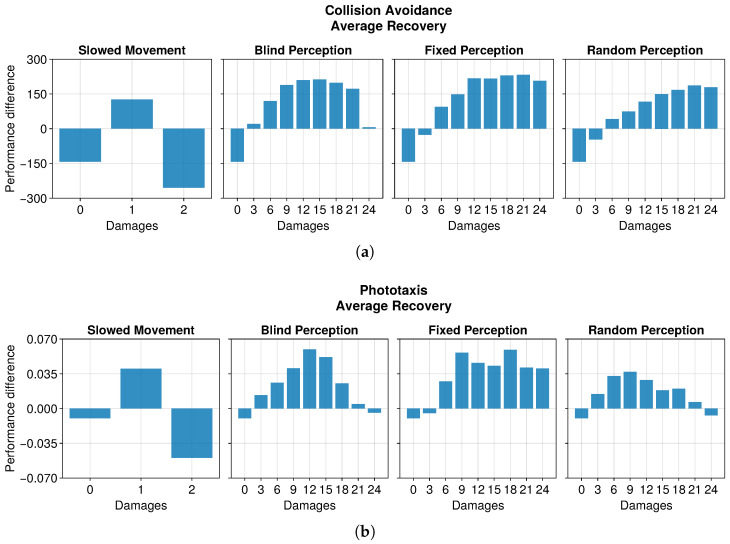
Average recovery of performance, calculated as the difference between the average performance at the end of the experiment and that of the exploitation epoch immediately after the occurrence of damage. This plot considers only replicas that learned the behavior without damages. (**a**) The recovery in the collision avoidance task. (**b**) The recovery in the phototaxis task.

In this section, we assess the capability of OA to recover the robot performance after a fault. We consider only the performance of the robots during the exploitation of the best solution, and we ignore the performance during exploration. The results show that OA was generally able to recover or maintain the robot performance after undergoing faults (see Figure 3). Nevertheless, we can distinguish the impact on the maximum recovered performance caused by different types of damage in each of the two tasks.

**Figure 4:**
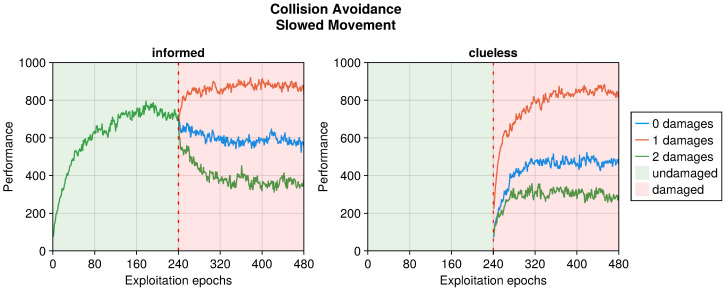
Performance according to the intensity of the damage on the actuators in the collision avoidance task. The trend is the average of the replicas that learned the behavior. Overall, a performance greater than 500 indicates a successful robot.

In the collision avoidance task, having a damaged actuator seemed to improve the performance (see Figure 4). The difference in the two wheels speed indeed tended to curve the trajectory of the robot, inducing a turn. Since the arena was a circuit, a turning behavior effectively navigated the environment. Normally, this is discouraged by the evaluation function, which penalizes unnecessary turns. However, this considers only if the motors are active, and not their effective rotation speed. The result is that the evaluation function we employed was not able to discriminate a turn due to a slowed motor from a straight movement, and thus could not penalize it. This enabled the robot to achieve a higher performance despite showing a suboptimal behavior. This hypothesis held when considering two faulty actuators. In this case, the robot no longer displayed the turning behavior. Instead, all its movements slowed down by half. This increased the time to perform a turn and move away whenever it encountered an obstacle, decreasing the performance with respect to the undamaged scenario. Additionally, OA did not recover the performance in the two-damages scenario. Instead, it decreased slowly until it stabilized.

**Figure 5:**
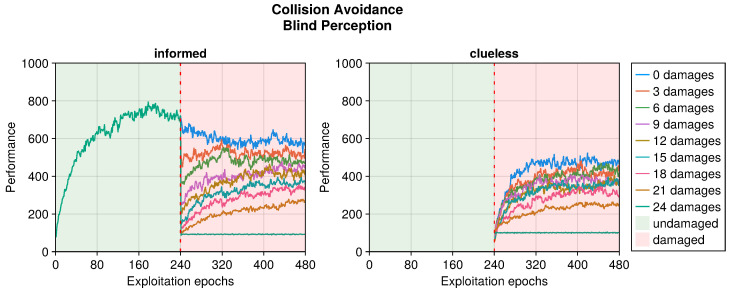
Performance according to the number of blind perceptions in the collision avoidance task. The trend is the average of the replicas that learned the behavior. Overall, a performance greater than 500 indicates a successful robot.

**Figure 6:**
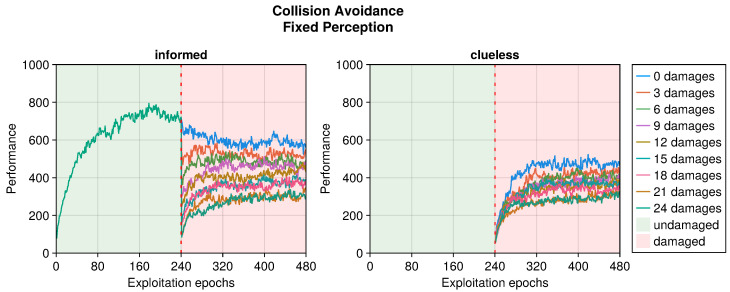
Performance according to the number of fixed perceptions in the collision avoidance task. The trend is the average of the replicas that learned the behavior. Overall, a performance greater than 500 indicates a successful robot.

**Figure 7:**
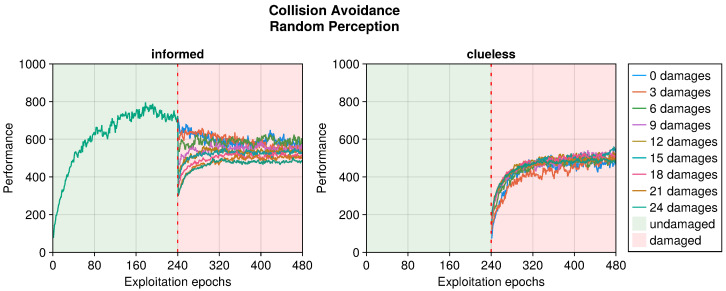
Performance according to the number of random perceptions in the collision avoidance task. The trend is the average of the replicas that learned the behavior. Overall, a performance greater than 500 indicates a successful robot.

Still referring to the collision avoidance task, the occurrence of sensory damages caused a sudden drop in the performance (see Figure 5, Figure 6 and Figure 7). Nevertheless, OA recovered the behavior in a short time regardless of the type of damage incurred. The differences occurred in the intensity of the performance drop immediately after the damage, and in the recovered performance. Specifically, blind robots experienced a substantial decrease with the slowest recovery (see Figure 5). Fixed perception caused a comparable drop in performance after damage but recovered faster (see Figure 6). Additionally, the performance attained after adaptation was slightly higher than that of blind robots, indicating a more manageable fault. Finally, random perception caused a reduced drop in performance with respect to the two other types of damage (see Figure 7). Additionally, the recovery was faster and led to a performance similar to that of the undamaged robot.

**Figure 8:**
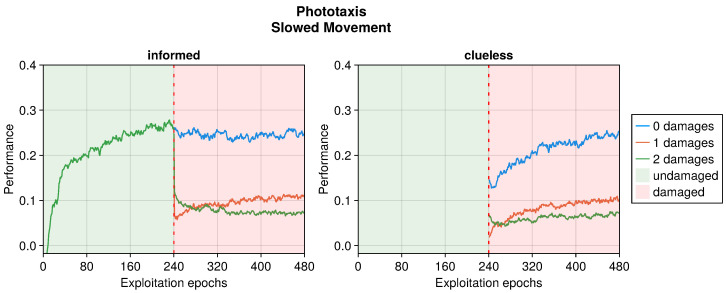
Performance according to the intensity of the damage on the actuators in the phototaxis task. The trend is the average of the replicas that learned the behavior. Overall, a performance greater than 0 indicates a successful controller.

In the phototaxis task, the peculiar trend displayed in the presence of damaged actuators was not replicated (see Figure 8). Indeed, the performance decreased gracefully according to the intensity of the fault. This is because, unlike the collision avoidance task, the phototaxis arena did not favor turning behaviors; instead, it required a straight trajectory toward the light to attain the highest evaluation. In the same way, a slower movement decreased the maximum traveled distance. This also limited the performance that the robot could attain, as it was bound to half of the maximum without damages. The results showed a modest recovery of performance in the case of just one instance of damage, with even a slight decrease in the case of two damages.

**Figure 9:**
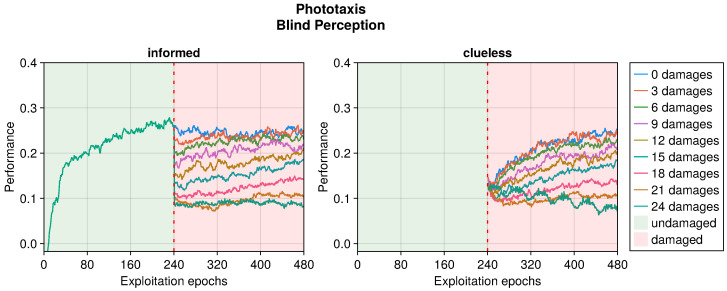
Performance according to the number of blind perceptions in the phototaxis task. The trend is the average of the replicas that learned the behavior. Overall, a performance greater than 0 indicates a successful controller.

**Figure 10:**
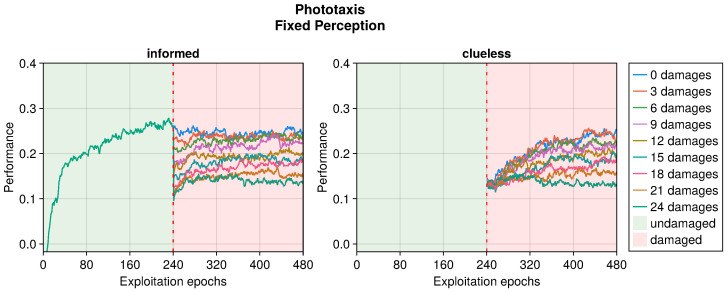
Performance according to the number of fixed perceptions in the phototaxis task. The trend is the average of the replicas that learned the behavior. Overall, a performance greater than 0 indicates a successful controller.

**Figure 11:**
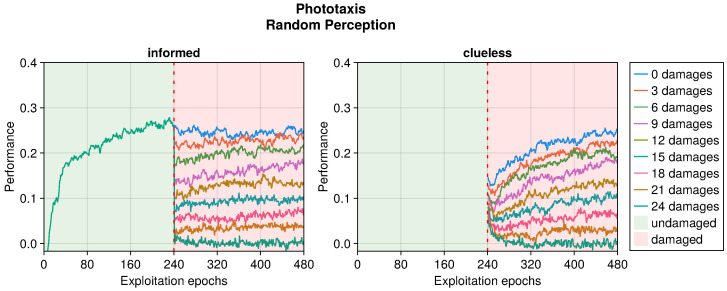
Performance according to the number of random perceptions in the phototaxis task. The trend is the average of the replicas that learned the behavior. Overall, a performance greater than 0 indicates a successful controller.

The performance trend in the phototaxis task with faulty sensors differed slightly from that in the collision avoidance task (see Figure 9, Figure 10 and Figure 11). When damages occurred, we still observed a drop in performance. However, this recovered more slowly throughout the entire duration of the experiment, often without stabilizing. Interestingly, with some degree of damage, the recovery even seemed enhanced. Indeed, slightly and heavily damaged robots recovered less and slower than moderately damaged ones. For instance, the recovery when subjected to 12 or 15 blind sensors was much greater than that with 3 or 24 faulty sensors. This trend was robust across all the experiments. The motivation is probably that a few damages cause a negligible drop in performance and therefore a limited possible recovery. Meanwhile, with many damages, recovering the performance becomes harder. A mean amount of damage causes a sensible drop but still permits recovering the performance to a great extent.

Even in the phototaxis case, we can distinguish the effect of different types of damage on the performance. With blind sensors, the performance drop was significant and did not notably recover for slightly or heavily damaged robots (see Figure 9). Differently, the recovery seemed to be at its maximum at approximately half faulty sensors. The experiments with fixed perception showed a similar trend, but even highly damaged robots were able to recover their performance (see Figure 10). Additionally, the recovered performance was greater than that in the case of blind robots, indicating a more manageable fault. Finally, with random perception, the drop in performance was the greatest and recovery was minimal (see Figure 11). Indeed, OA seems to be able to recover the performance only for slightly faulty robots. This differs from the results obtained in the collision avoidance task, where random perception was the least detrimental type of fault.

In addition to the trends just analyzed, we also investigated the effects of adaptation in “clueless” robots, which began damaged and with completely random input couplings (see Figure 4, Figure 5, Figure 6, Figure 7, Figure 8, Figure 9, Figure 10 and Figure 11). This permitted to compare the recovery time of “informed” robots that started with a previously working behavior, and to verify that this does not trap the controller into an ill solution. All experiments showed that the performance attained starting from a working controller (i.e., informed) was similar to that attained starting from a clueless robot. This suggests that adapting a working controller does not prevent a successful exploration of alternative solutions. Instead, it speeds up the search, attaining better performance soon after damage occurs. This is especially true for limited faults, which affect the performance less and thus require fewer modifications to become effective again. Additionally, this is especially true for the collision avoidance task, as in the phototaxis task the recovery speed-up was reduced.

## 4. Discussion

**Figure A1:**
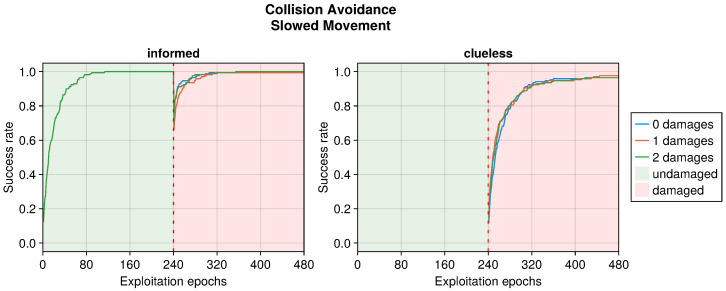
RLD according to the number of slowed motors in the collision avoidance task. This plot considers only replicas that learned the behavior without damages. Every performance greater than 500 is considered a success.

**Figure A2:**
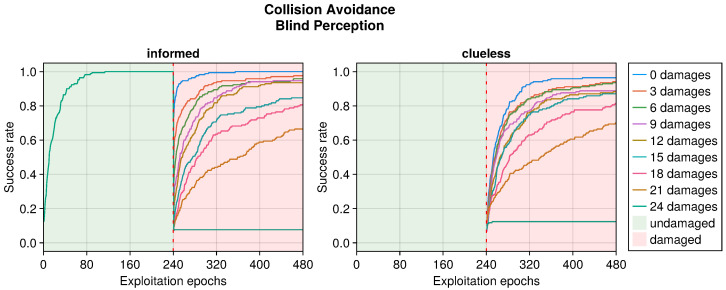
RLD according to the number of blind perceptions in the collision avoidance task. This plot considers only replicas that learned the behavior without damages. Every performance greater than 500 is considered a success.

**Figure A3:**
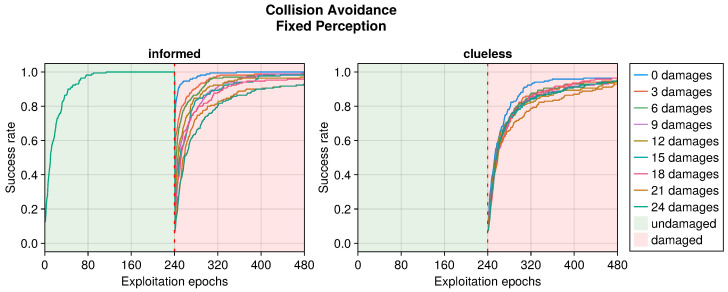
RLD according to the number of fixed perceptions in the collision avoidance task. This plot considers only replicas that learned the behavior without damages. Every performance greater than 500 is considered a success.

**Figure A4:**
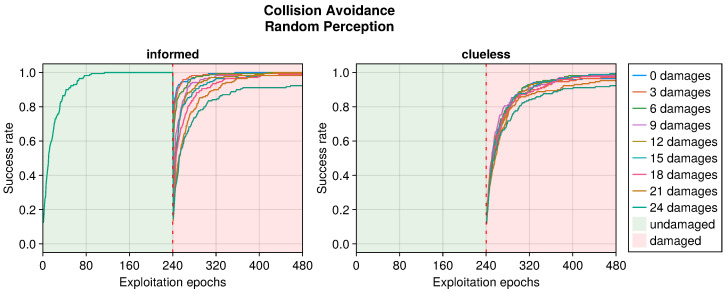
RLD according to the number of random perceptions in the collision avoidance task. This plot considers only replicas that learned the behavior without damages. Every performance greater than 500 is considered a success.

**Figure A5:**
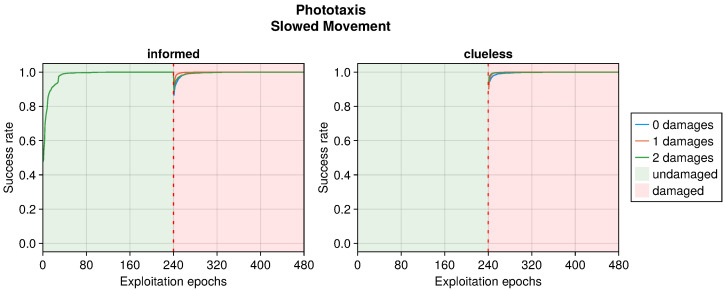
RLD according to the number of slowed motors in the phototaxis task. This plot considers only replicas that learned the behavior without damages. Every performance greater than 0 is considered a success.

**Figure A6:**
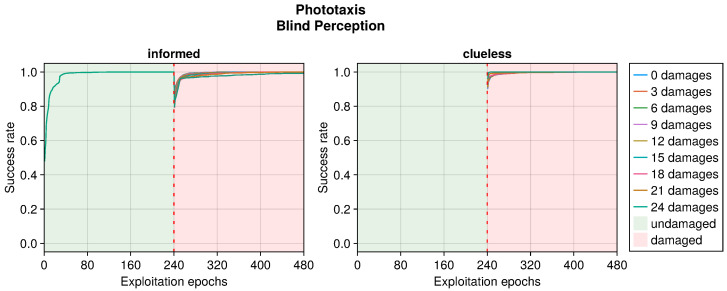
RLD according to the number of blind perceptions in the phototaxis task. This plot considers only replicas that learned the behavior without damages. Every performance greater than 0 is considered a success.

**Figure A7:**
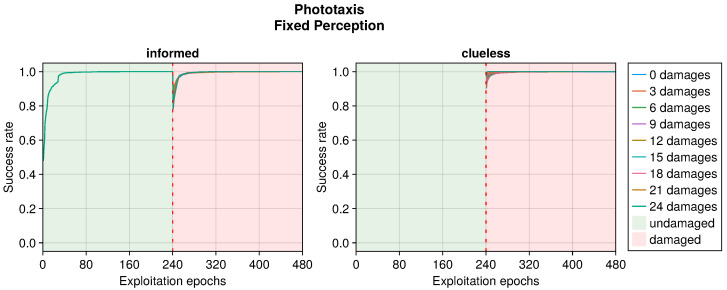
RLD according to the number of fixed perceptions in the phototaxis task. This plot considers only replicas that learned the behavior without damages. Every performance greater than 0 is considered a success.

**Figure A8:**
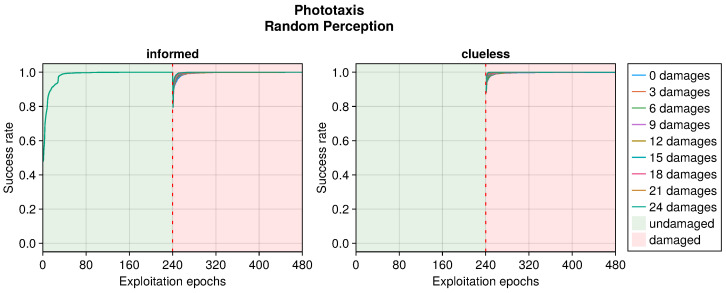
RLD according to the number of random perceptions in the phototaxis task. This plot considers only replicas that learned the behavior without damages. Every performance greater than 0 is considered a success.

**Figure A9:**
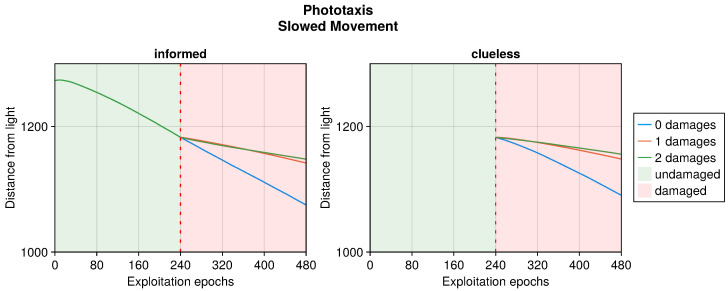
Robot distance from the light in time when subjected to slowed motion. This plot considers only replicas that learned the behavior without damages.

**Figure A10:**
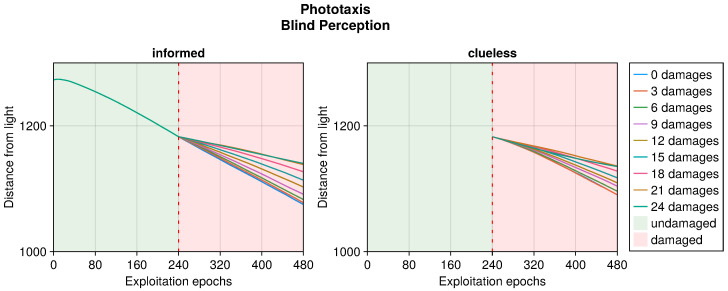
Robot distance from the light in time when subjected to blind perception. This plot considers only replicas that learned the behavior without damages.

**Figure A11:**
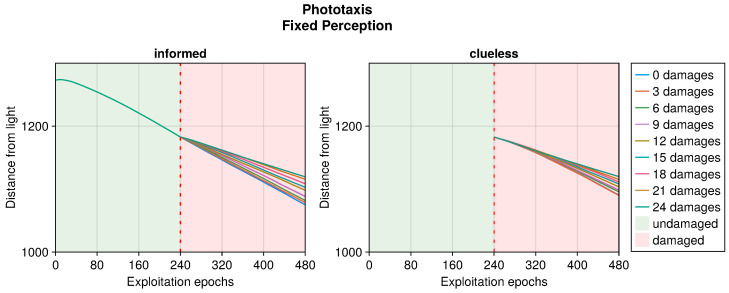
Robot distance from the light in time when subjected to fixed perception. This plot considers only replicas that learned the behavior without damages.

**Figure A12:**
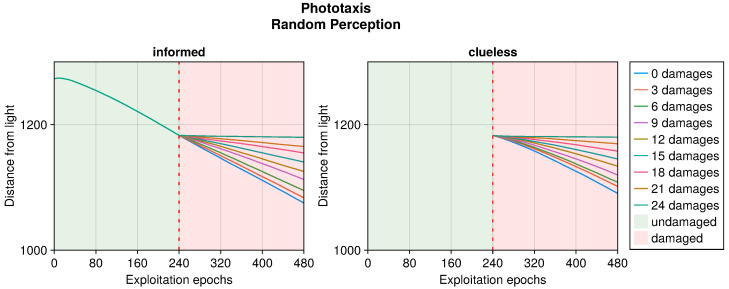
Robot distance from the light in time when subjected to random perception. This plot considers only replicas that learned the behavior without damages.

In this work, we investigated whether OA enables recovering the performance of a robot after the occurrence of damage and the time it requires. We found that OA is a valid strategy for overcoming damages in most situations (see Figure A1, Figure A2, Figure A3, Figure A4, Figure A5, Figure A6, Figure A7, Figure A8, Figure A9, Figure A10, Figure A11 and Figure A12). This permits to recover the performance to a degree proportional to the intensity of the fault. Indeed, more damages induced a greater drop in performance, which was usually followed by a recovery proportionally greater than that of fewer damages. Meanwhile, limited faults caused an almost negligible drop, which was soon recovered to a slightly better value. In the phototaxis task, this trend was slightly different, with heavily damaged robots recovering their performance less. We also identified how different types of damage produce different effects in the recovery. Specifically, we noticed that a fixed perception damage affected the robot less in the two tasks (i.e., the drop was limited and the recovery was fast). This is somewhat surprising, as we would expect a blind (or missing) perception to do so. The motivation is probably that the former enables modifying the dynamics of the BN in favor of the robot, eliciting the desired behavior in a specific environment. Not modifying the controller, blind perception removes the possibility to influence its dynamics and, therefore, to modify the intrinsic behavior of the robot. Random perception produced different results in the collision avoidance and phototaxis tasks. Specifically, it permitted attaining the highest recovered performance in the former, while it produced the worst behavior in the latter. The underlying cause of this difference remains unclear. Finally, damages to the actuation were generally the hardest to recover but could enhance the performance by exploiting the peculiarities of the evaluation function. While this does not actually imply an effective improvement in the generated behavior, it is still an interesting point to consider while devising the function itself. Interestingly, this peculiar trend has been observed even in other articles [12].

The aforementioned differences in the impact of different types of damage were not universal but depended on the environment in which the robot acted. Clearly, a robot with completely damaged sensors should not be able to act properly. Nevertheless, it succeeded in doing so thanks to OA, which exploited the peculiarities of the environment to select a suitable behavior. Similarly, when a robot was only partially damaged, OA permitted using both sensory perception and the peculiarities of the environment to improve the performance. This can be seen as a sort of overfitting of the behavior for a specific environment and for a specific type of damage. Usually, this would be considered negative, but due to the characteristics of OA, this is not particularly problematic. Indeed, the ability to generalize is useful for facing unexpected challenges, but it decreases in importance when we can directly adapt to them. Obviously, generalizing remains important, but it is no longer fundamental to succeed. All of these considerations suggest that the difference in the performance with different types of damages could arise from their synergy with the environment and by how easily OA exploits them. A notable example is that of one faulty actuator in collision avoidance, which curved the trajectory, simplifying the navigation of the circuit arena.

One interesting result is in the analysis of the time to recover the performance after the occurrence of damage. Indeed, trying to fix an already known controller might slow down the recovery, or even trap it in an unfavorable section of the search space. If this were the case, generating a new controller from scratch could lead to better results more quickly. Our results showed, instead, that modifying a known solution to fix the behavior led to a faster performance recovery with respect to learning from scratch. This even suggests that starting from known behaviors might speed up the discovery of controllers for different tasks.

One important aspect we want to highlight is the need to constantly re-evaluate the best solution [30]. Indeed, from preliminary experiments, we noticed that keeping only the best evaluation did not work in changing contexts. A “best controller” at time $t_{1}$ might not be the best at time $t_{2}$. If the maximum performance obtainable by the latter is lower than that achievable by the former, then the adaptation will keep searching for an impossible solution. Re-evaluating the best controller permits identifying when it loses effectiveness, and thus enables replacing it with a better one. Calibrating the re-evaluation weight (i.e., how much our valuation of the controller depends on its last assessment) finally determines the repulsion to change of the robot.

Just re-evaluating the controller is, however, not enough for an effective adaptation. The evaluation function itself has a primary role in the success of the performance recovery. Indeed, in order to work effectively, OA needs to properly assess the performance of the robot. Doing so requires that the evaluation function understands the quality of the behavior thanks to perception, internal, and/or external feedback. In this work, we imagined damages affecting the internal connections of the robot, from the sensors to the controller, and from the controller to the actuators. The result is that the robot can still evaluate its behavior and thus drive the adaptation to the desired outcome. Nevertheless, damages occurring on the sensors themselves would in that case affect even the capability of self-evaluation. Facing this problem, therefore, requires an evaluation system able to automatically identify inconsistent states [41]. Alternatively, it could rely on external evaluation or on multiple measures. Nevertheless, this aspect is out of the scope of this work, and thus it is not discussed here. Indeed, we only assessed OA as a mechanism to recover the performance in case of faults, finding it effective.

As the final point of this discussion, we want to highlight that the whole control system proposed and used in this work is extremely simple. This is because our goal is to start developing a mechanism able to operate on microscopic artificial agents. Obviously, this limits the complexity of the system we propose, which should be easily implementable even in hardware. This excludes the use of all the state-of-the-art mechanisms for fault recovery we know about, which are typically much more complex and computationally demanding with respect to the method we propose here. We claim that our controller fulfills the characteristics for straightforward hardware implementation, making it potentially suitable for adoption in microscopic robots. However, this constrained scenario prevents a direct comparison with other solutions existing in the literature.

## 5. Conclusions

In this work, we discussed the use of Online Adaptation to provide fault recovery to extremely simple and limited robots controlled by Boolean Networks. We explored its use in two tasks: collision avoidance and phototaxis. The results showed that OA is effective in recovering the performance of robots to a level similar to that before the damage. We found that adapting a previously working controller recovered the performance of robots faster than devising a new controller from scratch. Additionally, we found that larger damages caused a greater drop in performance but also permitted a greater recovery. We discussed the role of re-evaluation of the best controller as a necessary condition for OA to work. Finally, we proposed adaptation as a way to leverage the peculiarities of the environment to reach a desired goal more effectively.

Our approach focused on the performance recovery through an adaptation driven by an embedded evaluation function. Nevertheless, we did not consider the case in which the evaluation itself fails. Even though it was not the focus of this work, we recognize that this could limit the application of the proposed mechanism. Therefore, we intend to further explore this issue by investigating the best way to combine fault diagnosis with the fault recovery provided by OA.

## Data Availability

The code used during the experiments and the resulting data are available in publicly accessible repositories [39,40].

## Appendix A. Run Length Distribution

Plots of the Run Length Distribution (RLD), that is, the rate of replicas in which the robot attained a performance greater than a threshold in time.
